# Cellular and molecular mechanisms of drug dependence: An overview and update

**DOI:** 10.4103/0019-5545.33253

**Published:** 2007

**Authors:** Swapnil Gupta, Parmananda Kulhara

**Affiliations:** Department of Psychiatry, Post-Graduate Institute of Medical Education and Research, Chandigarh, India

**Keywords:** Drug dependence, cellular mechanism, receptors, neurotransmitters

## Abstract

Drug dependence is a major cause of morbidity and loss of productivity. Various theories ranging from economic to psychological have been invoked in an attempt to explain this condition. With the advent of research at the cellular and subcellular levels, perspectives on the etiology of drug dependence have also changed. Perhaps the greatest advance has been in the identification of specific receptors for each of the drugs, their target neurotransmitter systems and the intracellular changes produced by them. These receptors also provide potential targets for treatment strategies of drug dependence. This overview attempts to present the mechanisms in the development of dependence and the newer treatment strategies for the major drugs of abuse like alcohol, opioids, cannabis, nicotine and cocaine.

Human addictions are chronically relapsing disorders characterized by compulsive drug use, an inability to limit the intake of drugs and the emergence of a withdrawal syndrome during cessation of drug use. Dependence has been defined as a cluster of behavioral, cognitive and physiological phenomena that develop after repeated substance use. It typically includes a strong desire to take the drug, difficulties in controlling its use, persisting in its use despite harmful consequences, a higher priority given to drug use than to other activities and obligations, increased tolerance and sometimes a physical withdrawal state (International Classification of Diseases (ICD)-10).

Cellular biology is defined as the study of the physiology and biochemistry of intracellular processes. Molecular biology is the branch of biology that seeks to explain all biological processes in terms of genes and genetic changes. With the advent of neuroscience as an indispensable branch of biomedical research, there has been explosive growth in the biological understanding of the process of addiction at the cellular and molecular levels. In this update, we have described recent research findings so as to increase awareness regarding these aspects of drug dependence syndromes.

## NEUROBIOLOGY OF ADDICTION

Drug addiction has been conceptualized as a complex and chronic disease process occurring in the brain, which is modulated by genetic, developmental and environmental factors. The most consistent and reproducible finding in drug addiction is that abused substances activate the mesolimbic dopamine system, which reinforces both pharmacological and natural rewards. The mesolimbic system consists of dopaminergic neurons in the ventral tegmental area (VTA) and their axonal projections to terminal fields in the nucleus accumbens (NAc) and the prefrontal cortex.

Opioids, alcohol, nicotine, cannabinoids and psychostimulants all act on this system to increase synaptic levels of dopamine (DA). All these substances have specific receptors in the brain and the increase in dopamine levels in the mesolimbic system is the final effect that they produce. Receptor-mediated activity is the principal mechanism by which any chemical messenger acts. Chemical messengers are regulatory macromolecules, usually proteins. Receptors have two major functions of recognition and transduction. Correspondingly, each receptor has two domains, i.e., a ligand-binding and an effector domain. The ligand-binding domain has a hydrophilic and a lipophilic region and is usually heteropolymeric. The binding of the ligand causes a change in the quaternary structure of the receptor.

Receptors have various effector mechanisms, which are broadly of four types:

1. G protein-coupled receptors (Gs, Gi, Gq and G13)
2. Receptors with intrinsic ion channels
3. Enzymatic receptors
4. Receptors regulating gene expression

One of the most dramatic advances in drug abuse research has been the identification of the target of every major drug of abuse. This advance occurred with the advent of radioligand-binding techniques, the biochemical characterization of drug binding sites and ultimately, with the application of molecular biology to clone and isolate these structures. The various drugs of abuse and their respective receptor types are given in Table 1.

**Table 1:** Drugs of abuse and their target receptors

Drug	Receptor
Opiates	Agonists at mu, delta and kappa receptors (peptides)
Cocaine	Indirect agonist of dopamine by inhibiting its transporter
Nicotine	Nicotinic acetylcholine (Ach) receptors
Ethanol	Gamma aminobutyric acid (GABA) agonist and N-methyl-D-aspartic acid (NMDA) receptor antagonist
Amphetamines	Indirect agonist of dopamine by stimulating its release
Cannabinoids	CB1 and CB2 receptors

Drugs can upregulate or downregulate their receptors and their effector mechanisms [Table 2]. These changes are effected through genetic mechanisms and are implicated in the development of tolerance and withdrawal. Earlier biochemical data supported that the site of action of drugs was homogeneous. It is now known that there is great diversity in drug-receptor interactions. For example, nicotine was thought to have a homogeneous class of binding sites in the brain. It is now known that there are many different oligomeric receptors that bind and are activated by nicotine.

**Table 2:** Target receptors of drugs of abuse and effector mechanisms

Effector mechanism	Receptor
Gi (causing decrease in adenosine 3',5'-cyclic monophosphate (cAMP))	Muscarinic M_2_, dopamine D_2_, serotonin / 5-hydroxytryptamine 5-HT_1_, GABA_B_, CB_1_, opioid mil delta
Gq (causing activation of PlC)	D2, GABA_B_, Opioid kappa
G13 (channel regulation) Intrinsic ion channels	GABA_B_, Opioid mu and delta N_N_, NMDA, Kainate, GABA_A_

Both the diversity of the receptor types and the cross-modality of drug-receptor interactions are becoming more and more significant. It was earlier thought that drug use caused changes in the specific binding sites, inactivation mechanisms or levels of endogenous ligand. The diversity of drug receptors now forces a consideration of changes in the actual structure of the receptor molecule or changes in the distribution of these molecules on the surface of the neuron. Drugs of abuse also have long-term effects resulting from the expression of genes activated as a consequence of the action of the drug.

## OPIOID DEPENDENCE

Several mechanisms have been proposed to explain opioid dependence.

### cAMP hypothesis

Opioid receptors act by decreasing the activity of adenylyl cyclase and thereby decreasing intracellular adenosine 3',5'-cyclic monophosphate (cAMP) levels. This was discovered by Sharma *et al.*[1] when they demonstrated a decrease in intracellular cAMP levels after the addition of morphine to a culture of neuroblastoma cells. However, with continued exposure, cAMP levels return to normal and on addition of an opioid receptor antagonist, cAMP levels increased beyond control values. This showed that tolerance and dependence-like phenomena occurred at the cellular level and it was hypothesized that adaptations in the cAMP pathway contribute to opiate tolerance and dependence. This was called the cAMP hypothesis of opioid dependence. Chronic opioid exposure caused induction of adenylyl cyclase and protein kinase A and there was a sudden decrease in these enzymes on withdrawal of the opioid. It was also seen that all three opioid receptor types underwent tolerance development.[2] The mechanism of tolerance to kappa agonists was discovered to be the uncoupling of the receptor from the G-protein,[3] mediated by a beta adrenergic receptor kinase.[4]

### Changes in ion conductance

Activation of opioid receptors can affect membrane permeability to potassium ions. Activation of protein kinase C can attenuate opioid receptor activity and affect ion conductance.[5]

### Changes in endogenous ligands

Chronic morphine use causes feedback inhibition of endogenous opioid synthesis, which further leads to opioid dependence and withdrawal.[6] Opioid agonists have been shown to decrease the expression of proenkephalin messenger RNA (mRNA).[7] On the other hand, opioid antagonists increase the expression of proenkephalin mRNA or enkephalin peptides in some cell types. This implies that the opioid receptor has a direct or indirect effect on endogenous opioid genes.

### Plasticity in neuronal circuits

This occurs through antiopioid neurons such as neurons using nociceptin / orphaninFQ, glutamate, cholecystokinin or neuropeptide FF as neurotransmitters. A study demonstrated the loss of morphine analgesic tolerance in Nociceptin-orphanin (NOP) knockout mice. Naloxone-precipitated morphine withdrawal was markedly attenuated in NOP knockout mice.[8]

### The role of glutamate

Expression of gill glutamate transporter, gamma interferon-inducible lysosomal thiol reductase (GILT-1) mRNA was found to be increased in the striatum and NAc of morphine-dependent rats and decreased in morphine-withdrawn rats.[9] A glutamate transporter activator inhibited the development of physical and psychological dependence on morphine.[9]

### Other neurotransmitter systems

ET_A_ (endothelin A) receptor was involved in the development of neonatal morphine tolerance.[10] *In vitro* opioid withdrawal induced an opioid-sensitive cation current mediated by the GABA transporter-1 (GAT-1). GAT-1 may be a target for therapy to reduce withdrawal symptoms.[11] Substance P (SP) was also seen to modulate expression of opiate tolerance and withdrawal in rodents.[12]

## ALCOHOL DEPENDENCE

### The GABAergic system

Alcohol's effects on GABA-mediated chloride ion (Cl^-^) uptake into brain “microsacs” (membranes isolated from brain cells that form sealed bags) were studied and it was found that alcohol increased Cl^-^ uptake. Alcohol could thus enhance GABA-mediated inhibition of neurons.[13] Each GABA receptor consists of five subunits, which assemble to form a channel at the center of the complex.

Chronic alcohol administration had reduced GABA_A_ receptor function and lower levels of the GABA_A_ receptor antagonists were required to induce seizures. One-time alcohol intake enhanced GABA-induced Cl^-^ flow into mouse brain microsacs but no such effect occurred after chronic alcohol administration.[14]

Analyses in rats found that chronic alcohol treatment leads to reduced mRNA levels for one of the alpha subunits (*i.e*, the alpha_1_ subunit) as well as to decreased alpha_1_ protein levels.[14] These findings support the hypothesis that tolerance development involves reduced GABA_A_ receptor numbers.

### Glutaminergic system

Alcohol decreases the transmission at NMDA receptors and it was seen that the expression of certain subunits of NMDA receptors is increased in the cortex of ethanol-dependent individuals.[15] Alteration in the NMDA receptor function (assessed by response to ketamine) may contribute to a subjective response to ethanol and increase the risk of developing alcoholism.[16]

### Serotonergic system

Low levels of cerebrospinal fluid 5-hydroxyindoleacetic acid (CSF HIAA) have been associated with alcoholism especially with rapid onset, aggressiveness and severe impulsivity.[17–18] Specific serotonin reuptake inhibitors (SSRIs)-citalopram and fluoxetine are reported to decrease alcohol consumption.[19–21] Serotonin transporter density was lower in the cortex of alcoholic subjects (perigenual and anterior cingulate cortex).[22]

### Dopaminergic system

Chronic alcohol intake was associated with decreased mesostriatal dopaminergic activity in rodents and decreased levels of dopamine and its metabolites in alcoholic patients.[23] Decreased dopaminergic function led to compensatory adaptive changes of D2 receptors (hypersensitivity / increased density).[24] Alcohol-dependent subjects with early relapse showed low levels of DA and increased density of D2 receptors. This measurement has been proposed to be a biological marker of vulnerability to early relapse in patients with chronic alcoholism.[25] Association studies of neurotransmitter gene polymorphisms in Caucasians with alcoholism showed a significantly higher prevalence of a polymorphism of the D2 receptor gene (DRD2 TaqI B1 allele) in alcoholic subjects.[28]

### The endocannabinoid system

Chronic alcoholism causes a downregulation of CB1 receptors and their signal transduction system and increased the synthesis of the endogenous cannabinoids—arachidonylethanolamide and 2-arachidonylglycerol.[27] Deletion of the CB1 receptor has been seen to block voluntary alcohol intake in rats.[28] A CB1 receptor antagonist, SR141716 has also been shown to reduce alcohol consumption in rodents.[29,30]

### Glycine neurotransmitter system

Glycine receptors (GlyR) in the NAc might act as targets for alcohol in its mesolimbic DA-activating effect.[31] Glycine and strychnine alter extracellular dopamine levels in the NAc, probably via GlyR stimulation and blockade. Glycine and strychnine reciprocally alter alcohol consumption in ethanol high-preferring male Wistar rats.[32]

### Proteomics and alcoholism

Peroxiredoxin, creatine kinase, fatty acid binding protein are some of the proteins that are upregulated in chronic alcoholic patients. Synuclein, tubulin and enolases are downregulated. These proteins are associated with the neurodegeneration in chronic alcoholism and some of these overlap with the changes in Alzheimer's disease.[26]

## NICOTINE DEPENDENCE

### Cholinergic system

Nicotine acts on the nicotinic type of cholinergic receptors. Different combinations of alpha and beta subunits produce receptors with different responses to agonists and antagonists. Receptor sensitivities to agonists and antagonists are dependent upon the subunit composition of the receptor. The stimulation of nicotine receptors by nicotine shuts down the receptor by causing it to withdraw into a pit. Thus, dopaminergic stimulation of mesolimbic dopaminergic neurons stops soon after a low level of nicotine stimulation. Nicotine's effects are hence self-regulating and its effects on behaviour are less pronounced than cocaine. The number of binding sites changes with chronic exposure to nicotine.[5,33,34] Nicotine withdrawal in the ratis associated with upregulation of adenylyl cyclase activity in the amygdala. Adenylyl cyclase activity is stimulated by calcium / calmodulin (as is also the case for opioid and cannabinoid abstinence).[35]

### GABA and metabotropic glutamate receptors

2-methyl-6-(phenylethynyl)-pyridine (MPEP), a metabotropic glutamate receptor subtype 5 (mGluR5) antagonist, decreased nicotine self-administration in rats. Hence, compounds increasing GABAergic transmission and mGluR5 antagonists have the potential to be used as antismoking medications.[36]

### Opioidergic system

Nicotine withdrawal 24 h after nicotine cessation, caused a significant increase in preproenkephalin mRNA levels in the hippocampus and striatum. These effects were blocked by pretreating rats with mecamylamine. This suggests that brain opioid system(s) is / are involved in mediating nicotinic responses and its withdrawal.[37]

## COCAINE DEPENDENCE

### Monoaminergic systems

Cocaine is an inhibitor of monoamine transporters especially dopamine but also has minor effects at the serotonin and norepinephrine transporters. In a study by Hall in 2004,[7] it was seen that dopamine transporter (DAT) gene knockout (KO) mice continued to experience the rewarding effects of cocaine. So, mice with both serotonin transporter (SERT) gene and norepinephrine transporter (NET) gene knockouts were created. DAT / SERT KO eliminated cocaine reward and NET / SERT KO mice displayed increased cocaine reward.[7]

### Role of cannabinoids in cocaine seeking

The cannabinoid agonist, HU210, provokes relapse to cocaine seeking after prolonged withdrawal. Cannabinoid receptor antagonists prevent relapse to cocaine use.[38] The selective CB1 receptor antagonist, SR141716A, attenuates relapse induced by reexposure to cocaine-associated cues or cocaine itself.

### Effect on transcription factor FosB

Overexpression of FosB increases sensitivity to the locomotor-activating and rewarding effects of cocaine and morphine, increases cocaine self-administration and increases incentive drive for cocaine.[39]

## CANNABIS DEPENDENCE

Cannabis acts on the CB1 (central) and CB2 (on immune cells) cannabinoid receptors. CB1 receptors mediate inhibition of adenylate cyclase and calcium channels, stimulation of K^+^ channels and activation of mitogen-activated protein kinase. Acute effects of cannabinoids as well as the development of tolerance are mediated by G protein-coupled cannabinoid receptors.

Hepatic metabolism in tolerance to delta9tetrahydrocannabinol (delta9-THC) was tested by pretreating animals with SKF-525A (a microsomal enzyme inhibitor) or phenobarbital (a microsomal enzyme inducer). The data suggested (but did not demonstrate definitively) a metabolic mechanism of tolerance development. Lithium was found to prevent the cannabis withdrawal syndrome (increased expression of Fos proteins in oxytocin-immunoreactive neurons and increased oxytocin mRNA expression / increased oxytocin levels in the peripheral blood). The effects of lithium against the cannabinoid withdrawal syndrome were antagonized by systemic preapplication of an oxytocin antagonist.

The discovery of molecular mechanisms of drug dependence has led to the identification of ligands that can be reliable treatment options Table 3.

**Table 3:** Studies describing various receptor ligands as treatment options

Drug	As treatment for	Study
SR 141716A(CB1 antagonist)	Cannabis dependence	Rinaldi-Carmona *et al.,* 1994[40]
		Huestis *et al.*, 2001[41]
TRK 820 (kappa agonist)	Opioid and cocaine dependence	Hasebe *et al.*, 2004;[42]
		Heidbreder *et al.*, 1998[43]
Vigabatrin (GABA transaminase inhibitor)	Nicotine and cocaine dependence	Dewey *et al.*, 1999;[44]
		Brodie *et al.*, 2003[45]
MPEP (metabotropic Glu receptor antagonist)	Nicotine and cocaine dependence	Paterson *et al.*, 2003;[46]
BP 897 (D3 partial agonist)	Cocaine dependence	Pilla *et al.*, 1999[47]
CGP 444532 (GABA _B_ receptor agonist)	Nicotine and cocaine dependence	Brebner *et al.*, 1999[48]

## EMERGING TREATMENT STRATEGIES

18-Methoxycoronaridine (18-MC) decreases drug self-administration in several animal models. Thus, it may be a potential treatment for multiple forms of drug abuse (antagonist of alpha3beta4 nicotinic receptor).[49]

## CONCLUSIONS

The final common pathway of the action of drugs of abuse is through dopamine in the limbic system. Chronic administration of drugs leads to molecular changes in more than one neurotransmitter system and thus, various neurotransmitter systems are involved in the development of dependence on a single drug. Studying the neurobiological basis of addictive processes allows better understanding of current pharmacotherapy and will lead to the development of new and hopefully, more efficacious treatment strategies in the future.
